# Triple Therapy Protocol for Primary and Secondary Auricular Keloids: A Prospective Outcome Evaluation

**DOI:** 10.1097/DSS.0000000000003867

**Published:** 2023-07-03

**Authors:** Frank R. Datema, Joan Saridin, Ferdinand C.A. Timmer, Laura T. Rothuizen, Floris V.W.J. van Zijl

**Affiliations:** * Department of Otorhinolaryngology and Head and Neck Surgery, Erasmus Medical Center, Rotterdam, The Netherlands;; † Department of Oral and Maxillofacial Surgery, Erasmus MC, Rotterdam, The Netherlands;; ‡ Department of Otorhinolaryngology, Amphia Hospital, Breda, The Netherlands

## Abstract

**BACKGROUND:**

Ear keloid lesions present a significant challenge to the aesthetic surgeon. Keloids are known to recur and can cause severe cosmetic, functional, and psychological impairments. Several adjuvants to surgical removal have been promoted, with varying recurrence rates.

**OBJECTIVE:**

To evaluate the effectiveness of triple therapy to treat secondary (and large primary) auricular keloids.

**MATERIALS AND METHODS:**

Patients with secondary or large primary auricular keloids undergoing triple therapy were prospectively studied. Keloids were excised intramarginally under magnification and repeated triamcinolone acetonide 40 mg/mL injections were administered, followed by the application of a custom-made acrylate pressure device. Recurrent keloid formation and adverse events were monitored during a minimum of 6 months of follow-up.

**RESULTS:**

Sixteen auricular keloid lesions (3 large primary and 13 secondary) were subjected to the proposed technique with a mean follow-up of 28 months. All cases that adhered to the protocol were free of keloid after triple therapy. Side effects were limited to 1 case of lobular atrophy and slight hypopigmentation. All patients were satisfied with the results.

**CONCLUSION:**

The triple therapy protocol is highly effective in primary and secondary auricular keloid as long as patients remain compliant.

Trauma, inflammation, surgery, or burns of the skin can result in a variety of scar severity, ranging from fine line scars to hypertrophic and keloid scars. In contrast to hypertrophic scars, keloids rarely regress but continue to grow over time, spread beyond the margin of the original area of injury, can be painful or pruritic, and commonly recur after treatment. The pathophysiology of keloid is still not fully understood but considered as an impaired balance between fibroblastic proliferation and apoptosis as well as possible endothelial dysfunction.^1–3^ Keloid formation is unique to humans, especially those with a Fitzpatrick skin Type IV to VI. A family history is frequently present, although the mode of inheritance is unclear. Incidences of 4.5% to 16% have been reported.^4^ In the head and neck area, the external ear is most commonly affected and can cause severe cosmetic, functional, and psychological impairments.

Literature reports a variety of treatment modalities for auricular keloid, such as surgical excision, steroid injections, pressure therapy, silicone sheeting, cryotherapy, laser therapy, imiquimod cream, and radiotherapy. Presently, no universally effective method of treatment is favored, but any combination of modalities seems to benefit the high recurrence rate of surgery alone. The management of secondary keloids (arising after surgical resection of a primary keloid) is particularly challenging, as previous treatment has shown to increase the risk of recurrence.^5^

In this article, the results of protocolled triple therapy to treat secondary or large primary auricular keloids were described. The treatment protocol combines intramarginal keloid resection with steroid injections and pressure therapy with a custom-made acrylate device.

## Methods

This prospective study was conducted at the otorhinolaryngology department of an academic teaching hospital, from 2016 to 2021. Patients referred to the clinic for the management of primary or secondary auricular keloid were included. Exclusion criteria were patients with keloid locations unsuited for pressure therapy, such as the postauricular mastoid skin, and those unwilling to commit and comply to the treatment protocol. Informed consent for study participation was obtained from all patients, including the information that despite commitment, compliance, and an average treatment duration of 1 year, no guarantee could be provided for a permanent solution. After triple therapy, each patient was followed for at least 6 months to document recurrence.

### Triple Therapy Protocol

**Figure 1.**
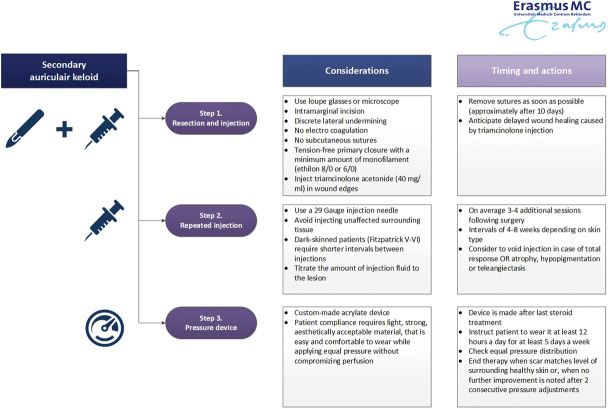
Flowchart of protocolled triple therapy for auricular keloids with considerations and clinical notes.

Our treatment protocol is adapted from Bran and colleagues^6^, and it combined intramarginal auricular keloid resection under magnification, with a sequence of 40 mg/mL triamcinolone acetonide injections and a custom-made acrylate pressure device (Figure 1).

#### Phase 1: Surgical Excision and Triamcinolone Acetonide 40 mg/mL Injection

Keloids were removed and debulked with an intralesional, bevelled excision under magnification. The residual keloid skin left at the margin was kept to a minimum. To further protect adjacent “keloid prone” skin and deeper tissue layers, undermining was minimized, and coagulation never performed. The thin keloid edges were tensionless approximated with a minimum of intradermal monofilament nonabsorbable sutures (Ethilon 8/0 or 6/0). After closure the wound was injected with a small amount of triamcinolone acetonide. Sutures were removed after 10 days to avoid dehiscence from delayed wound healing caused by the steroids.

#### Phase 2: Repeated Steroid Injections

Four weeks after surgery, the second steroid injection was administered using a 29-gauge tuberculin syringe with the amount of steroid titrated to the volume of the lesion (usually no more than 0.3 cc). Based on Fitzpatrick skin type and the therapeutic response, 3 additional steroid injections were performed, with 4- to 6-weeks intervals. If cases demonstrated an early full response (no visible and palpable residual keloid scar and no complaint of pain or itching) or side effects (atrophy, hypopigmentation, or telangiectasia), further steroid injections were avoided, and the treatment regimen was advanced to Phase 3.

#### Phase 3: Custom-Made Acrylate Pressure Device

**Figure 2.**
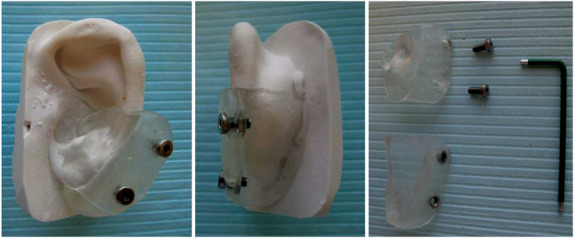
From left to right: Anterior part of the device with 2 nuts, posterior part of the device with bolts, overview of pressure set for the patient.

With the volume of the auricle now in a favorable condition, a silicone mold of the ear was made to extract a plaster duplicate (Figure 2). This duplicate facilitated the creation of a custom 2-part acrylate pressure device. The posterior part was polymerized including 2 or 3 small nuts and the anterior part with corresponding openings for the bolts. Care was taken to create a zone of equal pressure by the removal or addition of acrylate. Patients were trained how to assemble this ultralight device and instructed to wear it day and night for as many days as possible with a minimum of 12 hours a day for 5 days a week. During regular follow-up visits, the device was adjusted if needed by adding acrylate to guarantee sufficient pressure to the flattening tissue. Pressure therapy was terminated as soon as the scar matched surrounding healthy skin level or if no further improvement of scar level was observed.

## Results

Eleven consecutive patients with a total of 16 auricular keloid lesions were included in this study. Seven patients (63.6%) were female, and mean age at the initial presentation was 23.3 years (range, 11–46 years). The most common cause for keloid was piercing of the ear (*N* = 10; 62.5%), followed by otoplasty (*N* = 4; 25.0%) and auricular graft harvest (*N* = 2; 12.5%). Thirteen lesions (81.3%) were recurrences after a previous excision, and 3 (18.7%) lesions were primary. Mean follow-up was 28 months.

**Figure 3.**
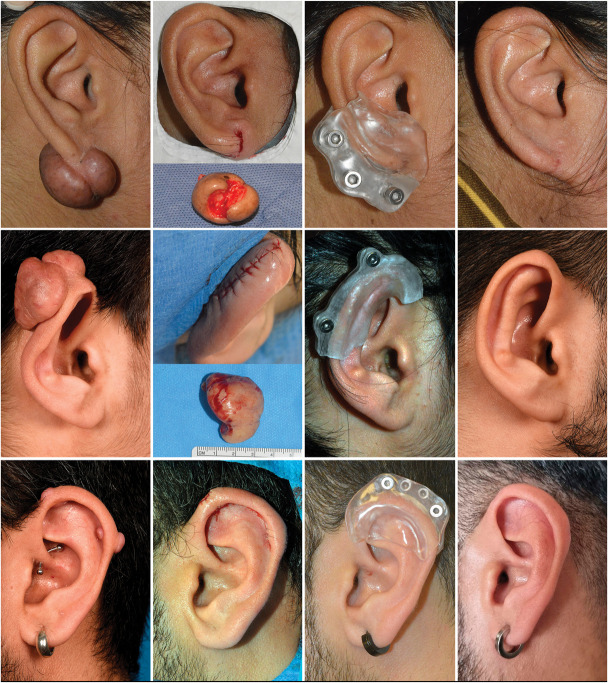
From top to bottom: Case #1, Case #5, and Case #8. From left to right: Initial presentation, direct postoperative situation, pressure device, and end result.

The number of triamcinolone injections varied from 2 to 5, and the duration of pressure therapy varied from 1 to 12 months. A complete and permanent response was encountered in 15 lesions (93.8%). Overall tolerance and compliance of the pressure device was good, with 2 patients requiring early adjustment to resolve pain and irritation from wearing the device. One patient (6.2%) had a recurrence of a lobular keloid. He refrained from wearing the pressure device due to a mechanical issue and was anxious to visit the hospital for refitting during the COVID-19 pandemic. He was retreated and until now has a complete response. Besides 1 case of atrophy of the lobule, no adverse events were encountered. An overview of results is provided in Table 1, and a presentation of 3 cases is given in Figure 3.

**TABLE 1.** Summary of 16 Cases Treated With Protocolled Triple Therapy

Case No	Sex/Age, y	Fitzpatrick Skin Type	Localization	Keloid Type	Initial Trauma/treatment	Number of Steroid Injections	Months Pressure Device	Result	Follow-up Time since Surgery, mo
1	Female/25	IV	Lobule	Secondary	Piercing/surgery	4	12	Complete response	15
2	Female/22	II	Antihelix	Primary	Piercing/none	5	3	Complete response	72
3	Female/17	III	Lobule	Secondary	Piercing/surgery and steroid injections	3	5	Complete response	26
4	Male/15	IV	Helix left	Secondary	Otoplasty/surgery and steroid injections	5	8	Complete response	47
5	Male/15	IV	Helix right	Secondary	Otoplasty/surgery and steroid injections	5	8	Complete response	47
6	Female/17	III	Helix	Primary	Piercing/none	5	1	Complete response	30
7	Female/24	III	Helix	Secondary	Piercing/surgery, steroid injections and radiotherapy	2	4	Complete response	32
8	Male/33	IV	Helix, multiple	Primary	Piercing/none	4	2	Complete response	35
9	Male/11	IV	Helix left	Secondary	Otoplasty/surgery	4	4	Complete response	10
10	Male/11	IV	Helix right	Secondary	Otoplasty/surgery	4	4	Complete response	10
11	Female/46	IV	Cavum conchae posterior left	Secondary	Graft harvest/surgery	5	12	Complete response	25
12	Female/46	IV	Cavum conchae posterior right	Secondary	Graft harvest/surgery	5	12	Complete response	25
13	Male/20	V	Lobule	Secondary	Piercing/surgery and steroid injection	4	1	Recurrence	8
14	Male/22	V	Lobule	Secondary	Piercing/surgery and steroid injection	4	4	Complete response	11
15	Female/24	V	Helix left, multiple	Secondary	Piecing/surgery	4	4	Complete response	13
16	Female/24	V	Helix right	Secondary	Piecing/surgery	4	4	Complete response	13

## Discussion

Scar formation is influenced by many factors, such as age, skin type, genetics, body part, excision method, closure technique, wound tension, and aftercare.^7^ Especially for hypertrophic scars and keloids, the challenge for the surgeon is to control as many of these factors as possible to reduce the risk of recurrence and to obtain a good aesthetic and functional result. Keloid recurrence rates after surgical removal alone range from 45% to 100%, illustrating the need for postoperative adjuvant treatment.^8–10^ Literature reports a variety of adjuvants, but evidence levels on the effectiveness of combined treatment modalities are low and recurrence rates are variable. A recent systematic review on recalcitrant auricular keloids reported recurrence rates as high as 17% after excision and pressure therapy, 25% after excision and brachytherapy, 53% after excision and steroid injections, and 56% after excision and external beam radiation.^11^

The primary aim of this study was to test the effectiveness of the triple therapy protocol for secondary auricular keloids, to avoid radiation therapy as a last resort. As described, triple therapy involves intramarginal resection under magnification, tensionless wound closure with a limited number of thin monofilament sutures, a sequence of steroid injections, and local pressure therapy with a custom-made acrylate device. A simplified rationale behind this combination is leaving a small rim of keloid scar is believed to avoid damage to the adjacent ‘keloid prone’ skin and to reduce tension during wound closure (tension is a stimulus for collagen synthesis). Intralesional steroid injections (triamcinolone acetonide 20 or 40 mg/mL) inhibit collagen synthesis and decreases collagenase activity.^12^ Local pressure therapy creates a zone of local hypoxia that is thought to cause fibroblast degeneration and subsequent collagen degradation. Using a variety of different auricular pressure devices, auricular keloid response rates of 90% to 100% are reported in the literature.^6,8,13–16^

The success of pressure therapy as an adjuvant to surgery and steroid injections relies on high-precise pressure adjustments and patient compliance. Patient compliance requires a light, strong, aesthetically acceptable and comfortable device that even for the youngest patients is easy to attach to the auricle. The customized pressure device as described in this article meets all these criteria. Functionally, the device needs to generate an equal zone of pressure that exceeds capillary pressure without inducing tissue necrosis. The reaction (blanching) of the skin and feedback of the patient (pain, discomfort) were used to decide if pressure adjustment was indicated.

The number of cases in this study is modest, which is related to the incidence and infrequent referral of keloids to the authors’ department. Nevertheless, the protocolled approach and strict follow-up of this unselected cohort provides us with valuable longitudinal outcome data. A complete response was encountered in 92.3% of secondary keloids and 100% in primary keloids. One could argue that triple therapy in primary auricular keloids is overtreatment. However, because reported recurrence rates of surgical excision and steroid injections range from 3% to 60%, it is felt that the addition of pressure therapy is a small investment to further reduce the chance of keloid recurrence in patients who are already in despair.

Despite low recurrence rates, triple therapy has limitations. First, you need the facilities and expertise to create and adjust a custom pressure device. Second, patients need to be fully committed to follow the “rather lengthy” protocol, which can easily take up more than 1 year. Third, a dedicated physician (assistant) is needed to warrant follow-up and treatment, especially when patients are inclined to drop out when the lesion seems under control during Phase 2 or early in Phase 3. As described, the 1 patient that did not complete the protocol, was the only 1 with a recurrence. It is important to realize that triple therapy is perhaps not suited for every busy medical practice. Finally, a physician needs to be aware that keloid patients can be in need for psychological assistance. Especially in case of recurrence and growth of the lesions, despair, loss of fate in medicine, aesthetic insecurity, avoidance of social activities, and depression can be encountered.

## Conclusion

Triple therapy shows good response rates in secondary (and primary) auricular keloids as long as patients remain compliant. Hence, triple therapy should be considered to less attractive adjuvants such as radiotherapy.
